# Family Practices' Achievement of Diabetes Quality of Care Targets and Risk of Screen-Detected Diabetic Retinopathy

**DOI:** 10.1371/journal.pone.0010424

**Published:** 2010-04-29

**Authors:** Martin C. Gulliford, Hiten Dodhia, Sobha Sivaprasad, Mark Ashworth

**Affiliations:** 1 Division of Health and Social Care Research, King's College London, London, United Kingdom; 2 Department of Public Health, Lambeth NHS Primary Care Trust, London, United Kingdom; 3 Ophthalmology Department, King's College Hospital, London, United Kingdom; Erasmus University Rotterdam, Netherlands

## Abstract

**Background:**

We aimed to determine whether family practices' achievement of diabetes quality of care targets is associated with diabetic retinal disease in registered patients.

**Methods:**

Data for achievement of diabetes quality of care targets, including the proportion of patients with HbA1c≤7.5%, for 144 family practices in London UK, for the years 2004/5 to 2007/8, were linked to data from a population-based diabetes eye screening programme collected from September 2007 to February 2009. Analyses were adjusted for age, sex, duration and type of diabetes, unadjusted diabetes prevalence, ethnicity and deprivation category.

**Results:**

Data were analysed for 24,458 participants with one or more eye screening results in the period. There were 9,332 (38%) with any diabetic retinopathy and 2,819 (11.5%) with sight threatening diabetic retinopathy (STDR), including 2,654 (10.9%) with maculopathy. Among participants registered at 13 family practices that were in the highest quartile for achievement of the HbA1c quality of care target for all four years of study, the relative odds of any diabetic retinopathy were 0.78 (0.69 to 0.88) P<0.001. For participants at 12 practices consistently in the lowest quartile of HbA1c achievement, the relative odds of any diabetic retinopathy were 1.16 (1.03 to 1.30), P = 0.015. In the highest achieving practices, the relative odds of maculopathy were 0.74 (0.62 to 0.89), P = 0.001 and STDR 0.77 (0.65 to 0.92), P = 0.004.

**Conclusions:**

The risk of diabetic retinopathy might be lower at family practices that consistently achieve highly on diabetes quality of care targets for HbA1c.

## Introduction

Maintaining and enhancing the quality of medical care is an increasing concern for all health systems. The US Institute of Medicine [1] drew attention to the ‘quality chasm’ that exists between the potential of modern medical management of chronic illnesses and the reality of routine chronic illness care. As a result, patients experience a considerable burden of preventable complications, and funders and providers of health services face rapidly escalating costs of chronic illness care.[2]

The use of incentives to encourage professionals to adhere to specific processes of care and achieve designated quality of care targets for intermediate outcome measures has received growing attention as one strategy to improve the quality of chronic illness care.[3] The English National Health Service has made systematic use of contractual financial incentives through a program for family practitioners known as the Quality and Outcomes Framework (QOF).[4] The program was introduced in 2004 to reward family practices for achieving clinical targets across a range of chronic conditions, including diabetes. Up to one-third of practice income may be derived from pay-for-performance incentives, with diabetes accounting for nearly 10% of all incentives. Several studies have demonstrated improving clinical performance under these new contractual arrangements, including increased levels of achievement of key process measures and intermediate outcomes.[5]–[8] Inequalities in care have also diminished.[9]

However, there remain significant doubts concerning the value of an approach based on quality of care targets. The system is costly and may emphasise only the more tangible aspects of patient care.[10] The apparent benefits may reflect improved recording of clinical information and it is not yet clear that these are translated into better patient outcomes. A key question concerns whether family practices' achievement of quality of care targets is associated with better health outcomes. In the present study, we aimed to determine whether family practices' consistent high-achievement of the HbA1c target was associated with the subsequent risk of diabetic eye disease. We linked data on practices achievement of pay-for-performance targets in the period 2004 to 2008, to the results of a population-based diabetes eye screening programme obtained in the period 2007 to 2009.

## Methods

The study was set in three inner-city boroughs in London UK that are characterised by high levels of social and material deprivation and have about one third of their total population drawn from black and ethnic minority groups. Family practices were included in the study if they were located in the three boroughs and contributed data to the Quality and Outcome Framework in all four years of study. Data for the achievement of QOF targets for diabetes were obtained from the NHS Information Centre as reported previously.[11] Data were analysed for the family practice-specific proportions of eligible diabetic patients in each year whose HbA1c was ≤7.5%, blood pressure was ≤145/85 mmHg or serum cholesterol was ≤5 mmol/l.[8] For each year of study, we grouped family practices into quartiles for achievement of each target. We then identified family practices that remained in the top quartile for achievement of HbA1c targets throughout all four years of study. We also identified those practices that remained in the bottom quartile of achievement of the HbA1c target throughout all four years of study. Similarly, we identified those practices that always remained in the top quartile for achievement of blood pressure or cholesterol targets. Data were also included for the prevalence of diabetes at each practice in 2007, based on the number of diabetes subjects included in the practice diabetes register and the practice list size. However, it was not possible to adjust diabetes prevalence for the varying distributions of age or ethnicity at different practices. Family practice level data for the achievement of targets were then linked to data for population-based diabetes eye screening using the National Health Service (NHS) general practice code. This code is unique to each family practice. The NHS general practice code was never missing from the Quality and Outcomes Framework data; neither was the NHS general practice code ever missing from any of the screening records. This is because the screening programme draws participants from general practice registers as the sampling frame.

The English diabetic retinopathy screening program is offered annually to all patients with diabetes who are registered with family practices in England.[12] In the three London boroughs included in this report, all diabetic patients registered with practices are offered appointments. Patients may also be referred by family practices or diabetes specialists. Screening is by 2-field digital photography. Photographs are primary and second-disease graded as recommended by the English National Screening Programme for Diabetic Retinopathy.[13] If there is a difference of classification, the images are referred for arbitration grade by consultant ophthalmologists. Screening outcomes were analysed using grades recorded separately for each eye. Participants were classified as not attending for screening if they were called for screening but had no recorded eye grade during the study period. Participants were classified as having maculopathy if the eye grade was recorded as M1 in either eye. Participants were recorded as having any diabetic retinopathy if the eye grading was recorded as R1, R2 or R3 in either eye. Sight-threatening diabetic retinopathy (STDR) is defined as any grade that includes severe non-proliferative retinopathy (R2), proliferative retinopathy (R3), and/or maculopathy (M1). Data for screening outcomes were analysed using the highest grades of eye disease recorded during the period.

Other fields included in analyses included age by ten year age group; sex; type of diabetes including Type 1, Type 2 and ‘Other and not specified’; duration of diabetes by five year group and a category for not known. The postcode of the subject's home address was linked to the Super Output Area (SOA) Indices of Multiple Deprivation 2007 (IMD) Score.[14] SOAs are small geographical units with resident populations of approximately 1,500 participants. The sample was divided into quintiles of deprivation using the ranks of the IMD scores. Self-assigned ethnicity recorded at the time of screening was analysed using the categories ‘African’, ‘Caribbean’, ‘Black other’, ‘South Asian’, ‘Mixed’, ‘Other ethnic group’ and ‘not known’.

Data were tabulated and random effects logistic regression models were fitted using Stata version 11.[15] Data were analysed at the individual participant level. Family practice was included as a random effect. In order to evaluate the stability of the estimates obtained we performed several sensitivity analyses. We allowed for the exclusion by practices of a small proportion of diabetic participants as ‘exceptions’. We corrected the estimated achievement of the HbA1c target for exceptions by multiplying the percent achievement by 100 minus the percent excluded as exceptions at each practice. Analyses were repeated using the exception-corrected values. We also repeated the analyses using the practices included in the highest or lowest tertiles rather than quartiles. We also repeated the analyses using estimation by Generalised Estimating Equations. The results of these sensitivity analyses are presented.

The Research Ethics Committee of Guy's Hospital, London reviewed the proposal for this project and advised that the project was a service evaluation and was not required to be ethically reviewed under the terms of the Governance Arrangements for Research Ethics Committees in the UK (Reference 2008–11; letter dated 29th July 2008). Data analysed for the project were derived from the minimum data set for the diabetic retinopathy screening programme. The proposal to access fully anonymised records was approved by the Caldicott Guardian of Guy's and St Thomas' Hospital (communication dated 18th June 2008).

## Results

**Figure 1:**
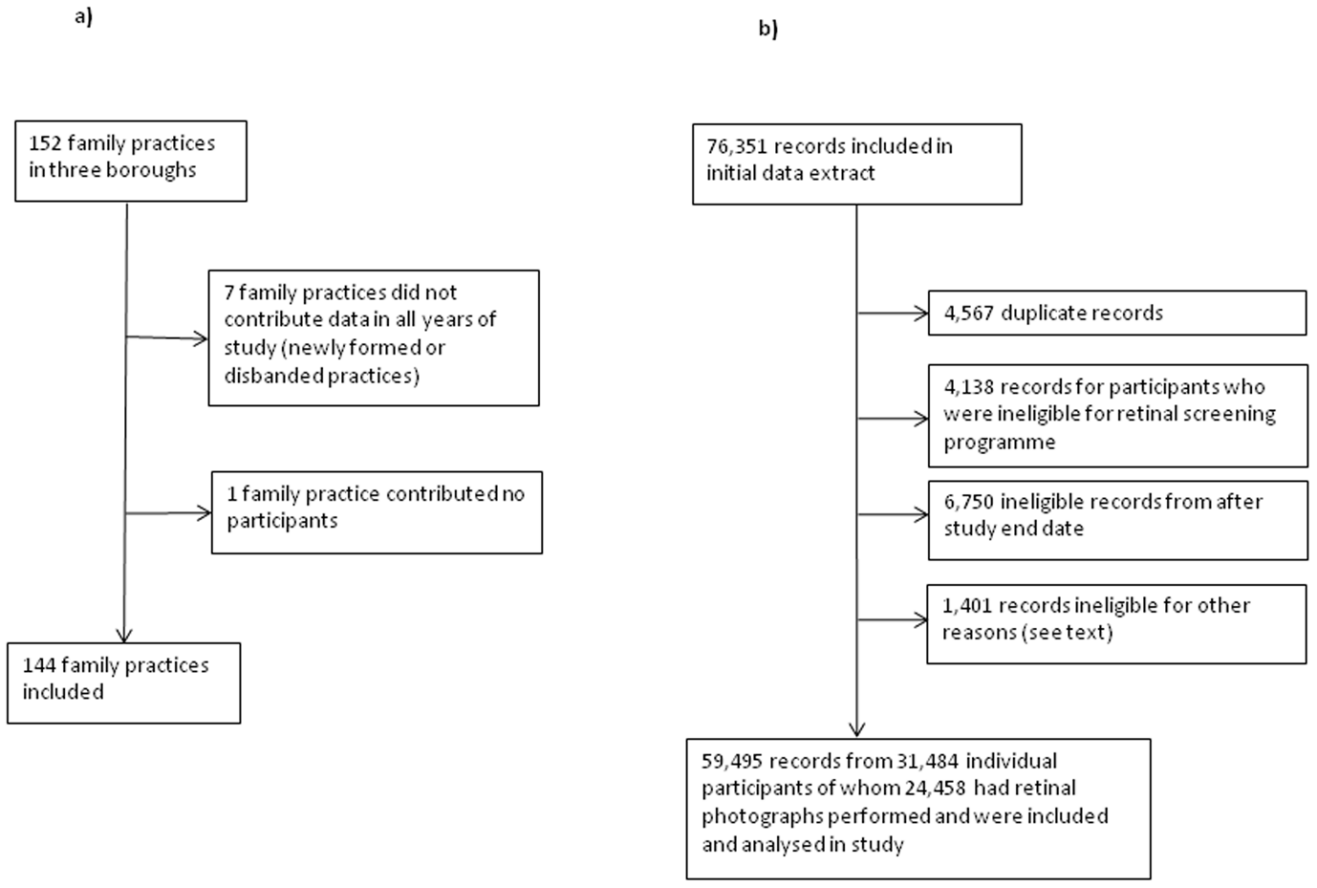
Flowcharts showing selection of family practices and screening records for analysis. Figure 1a): Selection of family practices. Figure 1b): Selection of records for analysis.

Figure 1 shows flowcharts detailing the inclusion and exclusion of family practices and screening records for the study. There were 152 family practices in the three boroughs during the period 2004 to 2008 but seven practices did not contribute data on pay-for-performance targets in each year of study and were excluded, as was one practice that had no participants included in the diabetes eye screening database, leaving 144 practices (95%) for further analysis. The 144 study practices identified 31,458 registered diabetic participants in their 2007–8 QOF returns. The median number of diabetic patients per practice was 178 (interquartile range 131 to 280). There were 36 practices in the highest quartile for HbA1c target in 2004–5 of whom 21 were also in the highest quartile in 2005–6, 17 in 2006–7 and 22 in 2007–8. There were 13 practices that remained in the top quartile for achievement of the HbA1c target throughout the period 2004 to 2008. There were 12 practices that remained in the bottom quartile for achievement of HbA1c target throughout this period.

Data were obtained from the Diabetes Eye Screening Program for all episodes from 1st September 2007 to 28th February 2009. Initially, data were evaluated for 76,351 records but 4,567 duplicate episodes were excluded; as were 4,138 episodes excluded as ineligible for screening because they attended a different hospital for eye screening (252), were deceased (2,630), were medically unfit (86), moved out of the area (925), were blind (27), denied being diabetic (65) , were under 12 years of age (10) or had opted out of the screening programme (143); 6,750 episodes with appointment dates after 28^th^ February 2008; 319 records excluded for participants that were not resident in South London boroughs, 1,003 from non-study practices; and 79 records with missing gender. There were then 59,495 records of appointments and episodes, from 31,484 participants, available for further analysis of whom 24,458 took up the screening offer and had retinal photographs performed.

Table 1 shows the characteristics of practices and individual patients included in the study groups. For practices that were always in the highest quartile for HbA1c achievement, the family practice-specific median (interquartile range) percent of patients achieving HbA1c≤7.5% was 66.7 (62.4 to 70.7) in 2004–5 increasing to 74.5 (72.7 to 80.6) in 2007–8. For practices that were always in the lowest quartile, the equivalent figures were 40.7 (38.4 to 43.9) in 2004–5 increasing to 52.5 (48.9 to 55.7) in 2007–8. In 2007, median achievement of the blood pressure target for the higher group of practices was 87% compared with 68% for the lower group of practices; for the cholesterol target the median achievement was 87% and 71% for higher and lower performing practices respectively. Higher achieving practices were generally similar to all practices but they showed a slightly higher rate of exception reporting than all practices. Practices that were low achieving for the Hba1c target showed lower screening uptake, a higher proportion of ethnic minority patients and a higher proportion of registered diabetic patients resident in the most deprived areas. Individual patient deprivation score ranks were correlated within practices, with an intraclass correlation coefficient of 0.28.

**Table 1:** Characteristics of participants according to pay-for-performance category.

			Achievement of HbA1c target			
		All practices	Practices always in top quartile 2004–2008	P value^a^	Practices always in bottom quartile 2004–2008	P value^a^
**Practice-level data**						
Practices		144	13		12	
Diabetes patients per practice (median, IQR)^b^		178 (131 to 278)	190 (157 to 233)	0.300	166 (109 to 302)	0.969
Diabetes prevalence (median, IQR, %)		3.4 (2.9 to 4.1)	3.0 (2.4 to 3.7)	0.116	3.7 (3.1 to 4.6)	0.198
Percent excepted from HbA1c target in 2007 (median, IQR)^b^		10.8 (7.6 to 14.4)	12.1 (7.7 to 19.5)	0.021	9.8 (6.7 to 15.6)	0.945
Percent of registered patients achieving HbA1c≤7.5% (median, IQR)^b^	2004	53.3 (46.5 to 59.4)	66.7 (62.4 to 70.7)		40.7 (38.4 to 43.9)	
	2005	56.8 (50.0 to 63.3)	79.4 (72.0 to 80.8)		46.5 (40.4 to 48.4)	
	2006	63.2 (55.9 to 70.0)	74.4 (72.3 to 83.9)		50.9 (46.9 to 53.1)	
	2007	63.6 (58.5 to 68.2)	74.5 (72.7 to 80.6)		52.5 (48.9 to 55.7)	
**Patient-level data**						
Called for screening		31,484	2,440		2,668	
Screened		24,458 (78)	1,955 (80)	0.046	1,948 (73)	0.001
Female^c^		11,966 (49)	952 (49)	0.859	964 (49)	0.576
Type 1^c^		1,571 (6)	144 (7)	0.254	106 (5)	0.174
Diabetes duration ≥10 years^c^		8,078 (26)	668 (27)	0.140	646 (24)	0.574
Ethnic minority group^c^		10,353 (42)	809 (41)	0.814	1,140 (59)	0.001
Most deprived quintile^c^		4,863 (20)	334 (17)	0.442	561 (29)	0.029
Least deprived quintile^c^		4,952 (20)	497 (25)	-	212 (11)	-

Figures are frequencies (percent) except where indicated.

a test for difference between category and practices or patients not in that category.

b figures are median (IQR) for distribution of practice-specific proportions.

c figures are frequencies and percent of those who were screened for diabetic eye disease.

Table 2 shows the distribution of any diabetic retinopathy, maculopathy and sight threatening diabetic retinopathy for practices in relation to achievement of pay-for-performance targets over time. Odds ratios were adjusted for age group, sex, type of diabetes, duration of diabetes, self-reported ethnic group and deprivation quintile as well as each of the variables shown. For practices that always remained in the highest quartile for HbA1c achievement, the prevalence of any diabetic retinopathy was approximately 4% lower, with maculopathy 2% lower and STDR 2% lower, than for other practices. In adjusted analyses, the relative odds of diabetic retinopathy were approximately 22% lower for high achieving practices than for other practices. The estimated adjusted relative odds for maculopathy and STDR were also lower for practices that achieved highly on HbA1c targets than other practices. For practices that were always in the lowest quartile for HbA1c achievement the prevalence of any diabetic retinopathy was approximately 3% higher, and the adjusted relative odds of any diabetic retinopathy were about 16% higher than for other practices. Similar point estimates were observed for maculopathy or STDR, but associations for these outcomes did not reach conventional levels of statistical significance. There was no association of diabetic retinopathy with consistently high achievement of the blood pressure target or the cholesterol target.

**Table 2:** Association of practices' achievement of targets for HbA1c, blood pressure or cholesterol with any retinopathy, sight threatening retinopathy and maculopathy.

Achievement of targets from 2004/5 to 2007/8				Any diabetic retinopathy (R1, R2, R3)			Maculopathy (M1)			Sight-threatening retinopathy (M1, R2, R3)		
		Practices	Participants	n (%)	Odds ratio (95% CI)^a^	P value	n (%)	Odds ratio (95% CI)^a^	P value	n (%)	Odds ratio (95% CI)^a^	P value
Always highest quartile for HbA1c target	Yes	13	1,955	673 (34)	0.78 (0.69 to 0.88)	<0.001	172 (9)	0.74 (0.62 to 0.89)	0.001	188 (10)	0.77 (0.65 to 0.92)	0.004
	No	131	22,503	8,659 (38)			2482 (11)			2,631 (12)		
Always lowest quartile for HbA1c target	Yes	12	1,948	790 (41)	1.16 (1.03 to 1.30)	0.015	256 (13)	1.13 (0.98 to 1.32)	0.107	267 (14)	1.12 (0.96 to 1.30)	0.141
	No	132	22,510	8,542 (38)			2398 (11)			2,552 (11)		
Always highest quartile for blood pressure target	Yes	11	1,646	621 (38)	1.00 (0.88 to 1.14)	0.964	190 (12)	1.08 (0.90 to 1.29)	0.406	199 (12)	1.05 (0.88 to 1.25)	0.587
	No	133	22,812	8,711 (38)			2464 (11)			2,620 (11)		
Always highest quartile for cholesterol target	Yes	10	1,651	613 (37)	1.02 (0.89 to 1.17)	0.743	176 (11)	1.01 (0.84 to 1.22)	0.917	189 (11)	1.02 (0.85 to 1.23)	0.801
	No	134	22,807	8,719 (38)			2,478 (11)			2630 (12)		

Figures are frequencies (row percent) except where indicated.

a odds ratios were adjusted for age group, sex, type of diabetes, duration of diabetes, unadjusted diabetes prevalence in 2007, self-reported ethnic group and deprivation quintile as well as each of the variables shown.

In a one-way analysis of variance, there was evidence of practice-level variation in the distribution of retinopathy. Intraclass correlation coefficients (ICCs) by practice were: for diabetic retinopathy 0.007 (P<0.001), for maculopathy 0.003 (P<0.001) and for sight-threatening retinopathy 0.001 (P = 0.003). These small ICCs may be interpreted as showing evidence of practice-level variation in the three measures of retinal disease. The ICCs were of small magnitude suggesting that within practices the distribution of retinal disease is, as expected, largely determined by individual characteristics such as the duration of diabetes and quality of blood glucose control in each subject.

Table 3 shows the results of sensitivity analyses. Correction for practices' exclusion of certain patients as exceptions tended to increase the strength of estimated associations. Use of tertiles, rather than quartiles, for analysis tended to diminish observed associations but did not eliminate them. There were 20 family practices (14%) always in the top tertile, and 17 (12%) of practices always in the bottom tertile, for HbA1c achievement. Use of alternative statistical methods for estimation did not alter conclusions. No association with any diabetic retinopathy was observed for family practices that were in the highest or lowest quartiles for only two or three years of study.

**Table 3:** Results of sensitivity analyses in which estimation methods and assumptions were varied.

	Any diabetic retinopathy			
	Highest performing 2004–8		Lowest performing 2004–8	
	OR (95% CI)^a^	P value	OR (95% CI)^a^	P value
Highest and lowest quartiles defined after correcting for exception reporting	0.75 (0.64 to 0.89)	0.001	1.20 (1.07 to 1.36)	0.003
Highest and lowest tertiles used instead of quartiles	0.86 (0.77 to 0.96)	0.005	1.12 (1.01 to 1.23)	0.026
Estimation using Generalised Estimating Equations	0.78 (0.69 to 0.89)	<0.001	1.16 (1.04 to 1.29)	0.007
Association for practices that were highest/lowest quartile for two or three years only	1.02 (0.94 to 1.12)	0.612	1.01 (0.93 to 1.10)	0.746

(OR, odds ratio; CI, confidence interval).

a odds ratios were adjusted for the same variables as in Table 2.

## Discussion

These results suggest that diabetic patients who are registered with family practices that consistently achieve highly on targets for HbA1c have a reduced risk of retinopathy. This association is biologically plausible. In type 2 diabetes, a 1% reduction in mean HbA1c in type 2 diabetes is associated with a 37% reduction in risk of microvascular complications.[16] In type 1 diabetes, differences in HbA1c explain ‘virtually all’ of the risk of microvascular complications of diabetes.[17] Although the association between HbA1c and retinopathy is well established [18], few studies have identified organisational characteristics that may mediate this relationship. The association between HbA1c achievement and retinopathy was graded, with practices that are consistently in the lowest quartile for achievement of the HbA1c target showing an increased risk of retinopathy. We did not find a large effect. However, four years is a short space of time in the evolution of diabetic retinopathy and the detection of any association may be clinically important. The study had the strength of the large sample size required to detect small effects.

The association between HbA1c achievement and retinopathy was specific. Associations were not observed for achievement of blood pressure or cholesterol targets. Lower blood pressure is associated with a reduced rate of progression of diabetic retinopathy. In the UK Prospective Diabetes Study, tight blood pressure control was associated with a 34% reduction in retinopathy progression over nine years.[19] High achievement of blood pressure targets might be expected to be associated with reduced retinopathy but this was not observed in these analyses. One possible explanation may be the generally high level of achievement of blood pressure targets in the Quality and Outcomes Framework. In England, the blood pressure target was achieved for median 71% of diabetic patients in 2004–5 and 80% in 2007–8.[8] In our data, the top performing practices had 87% achievement of the blood pressure target in 2007, compared with 77% for all other practices and 68% for the lowest performing practices. These figures indicate a higher overall achievement, and slightly smaller disparities, for the blood pressure target as compared to the HbA1c target.

We evaluated the achievement of quality of care targets during a four-year period before the evaluation of diabetic retinopathy. Nevertheless, we cannot determine whether the observed differences in the frequency of retinopathy were not already present before the introduction of pay-for-performance. In common with other studies that have evaluated the English pay-for-performance program [5], [9], our study did not have the benefits of a control group and allocation through randomisation. It is therefore important to employ appropriate caution in drawing possible causal inferences. We cannot prove that the pay-for-performance incentives were the cause of lower HbA1c values at study practices and the findings may not be applicable outside the context of UK primary care. Nevertheless, the results suggest an important overall conclusion; family practices that on aggregate achieve better blood glucose control, whatever its cause, may experience less diabetic retinopathy in their patients.

Studies have shown that quality of primary care is associated with socio-economic position [20] but in the UK inequalities in diabetes care are becoming less consistent [21], [22]. Residual confounding might be advanced as an explanation for the observed association between pay-for-performance achievement and diabetic retinopathy. High-achieving practices served slightly less deprived populations, and low-achieving practices served substantially more deprived populations on average than other practices. Low socio-economic position and deprivation have generally been shown to be associated with increased frequency of diabetes-related complications.[23] However, the evidence with respect to diabetic retinopathy is conflicting with some studies showing no association with socioeconomic position [24], [25] or giving inconsistent results [26]. While our analyses were adjusted for deprivation score, residual confounding might have been present as deprivation was measured at small-area level rather than being based on individual subject characteristics. The specificity of the association for achievement of the HbA1c target and the lack of association with the blood pressure or cholesterol target argue against this interpretation. Socio-economic position was more strongly associated with poor performance than with high performance, but retinopathy was less strongly associated with poor performance and more strongly associated with high performance. However, elevated HbA1c may be part of the causal pathway linking lower socio-economic position to retinopathy. Socioeconomic position is not a true confounder. Even if targets are more readily achieved by practices serving more affluent populations, this does not vitiate the conclusion that the resulting better control of blood glucose may contribute to lower risk of retinopathy.

An additional explanation for the association of target achievement with diabetes eye screening outcomes is the socio-economic patterning of screening uptake. In particular, screening uptake was lower at low-performing practices and screening uptake may be differential with respect to the risk of retinopathy. However, a previous study found only modest socio-economic inequalities in diabetes eye screening outcomes in the study area.[25], [27]

Ascertainment of early diabetes might be greater at better performing practices, leading to a higher prevalence of diabetes and apparently lower frequency of retinopathy. Our analyses were adjusted for diabetes prevalence. However, at the level of the family practice, distributions of age or ethnicity may have an important influence on diabetes prevalence and our estimates for diabetes prevalence were not adjusted for these variables.

Individual-level data for diabetic retinopathy grade were clustered by family practice as evidenced by the positive intraclass correlation coefficients for retinal disease. This was recognised in analyses in which random effects logistic models were used to inflate standard errors for clustering. Additional analyses were implemented using either Generalised Estimating Equations. Either of these analytical approaches gave results that were generally consistent with those presented. However, the small magnitude of the ICCs suggests that even at higher- or lower-performing practices, individual level characteristics such as the duration of diabetes or the quality of blood glucose control in each subject are, as expected, crucial in determining the distribution of diabetic retinal disease.

Data for HbA1c were aggregated to practice level and only referred to the achievement of a single target of ≤7.5%. Estimated associations were somewhat sensitive to whether quartiles or tertiles were used. We used an approach to modelling the time dimension that required practices to have remained in the highest or lowest quartile of performance over four years. A more sophisticated approach to analysis might be to model the achievement of the HbA1c target as a continuous variable in repeated measures framework. However, as there are as yet only four time points represented in the dataset, accumulation of further years' data would provide suitable material for such an analysis.

The present data provide evidence that family practices that on aggregate achieve highly on the HbA1c quality of care target over a period of at least four years, may have lower risk of retinopathy among their registered diabetic patients. This supports the notion that initiatives to promote quality of care, with targets for intermediate measures, may be associated with improved long-term health outcomes. Practices that remained in the highest quartile for fewer than four years did not show an association with retinopathy. This draws attention to the potential importance of longer term time horizons than the annual review cycle currently incentivised in the pay-for-performance program. Consistent achievement of targets over longer periods than one year may merit particular attention. We acknowledge a number of limitations to the interpretation of the present data, nevertheless our study demonstrates the potential of data linkage in the future evaluation of policy interventions. Future analyses should aim to provide additional information by utilising data collected over longer periods of time, and by evaluating incident eye disease both before and after the introduction of quality of care initiatives.
